# Toward a therapy for mitochondrial disease

**DOI:** 10.1042/BST20160085

**Published:** 2016-10-19

**Authors:** Carlo Viscomi

**Affiliations:** 1 MRC Mitochondrial Biology Unit, Wellcome Trust/MRC Building, Hills Road, Cambridge CB2 0XY, U.K.; 2 Molecular Neurogenetics Unit, The Foundation ‘Carlo Besta’ Institute of Neurology, IRCCS, Milan 20133, Italy

**Keywords:** gene therapy, mitochondria, mitochondrial dysfunction, mouse models, therapeutics

## Abstract

Mitochondrial disorders are a group of genetic diseases affecting the energy-converting process of oxidative phosphorylation. The extreme variability of symptoms, organ involvement, and clinical course represent a challenge to the development of effective therapeutic interventions. However, new possibilities have recently been emerging from studies in model organisms and awaiting verification in humans. I will discuss here the most promising experimental approaches and the challenges we face to translate them into the clinics. The current clinical trials will also be briefly reviewed.

## Introduction

The main function of mitochondria is to convert the energy derived from nutrients into heat and ATP, a high-energy molecule exploited by the cell biochemical machineries. This process is carried out by the respiratory chain (RC) through oxidative phosphorylation (OxPhos) [1]. Respiration is performed by four multiheteromeric RC complexes, cI–IV, that transfer electrons from the NADH and FADH_2_, generated by intermediate metabolism, to molecular oxygen. Mammalian mitochondria have their own multicopy DNA (mitochondrial DNA, mtDNA), which encodes 13 subunits of the RC complexes I, III, IV, and V (complex II is only composed by 4 nucleus-encoded subunits), 22 transfer RNA, and 2 ribosomal RNA.

Mitochondria are double-membrane organelles, with the inner membrane folded into cristae where the respiratory complexes are housed. The electron flow is coupled to the translocation of protons across the inner mitochondrial membrane generating an electrochemical gradient, which is then exploited by RC complex V (cV or ATP synthase) to carry out the condensation of ADP and Pi into ATP [2].

Beside mtDNA-encoded proteins, the vast majority of the ∼1500 polypeptides forming the mitochondrial proteome is encoded by nuclear genes, which are translated in the cytosol into proteins and finally imported into the organelles. These proteins are required for a massive number of biological processes, such as replication, transcription, and translation of the mtDNA, formation and assembly of the RC complexes, fission–fusion of the mitochondrial network, signaling, and execution pathways (e.g. ROS production and apoptosis) [1].

Primary mitochondrial diseases can be attributed to mutations in both mitochondrial and nuclear genomes [3]. MtDNA mutations include homo- or heteroplasmic point mutations and heteroplasmic large-scale rearrangements. Examples of classical mtDNA-related diseases are mitochondrial encephalomyopathy with lactic acidosis and stroke-like episodes (MELAS), myoclonic epilepsy with ragged red fibers, neurogenic weakness, ataxia, and retinitis pigmentosa (NARP), Leigh syndrome (LS), Leber's hereditary optic neuropathy (LHON), sporadic progressive external ophthalmoplegia, Kearns–Sayre syndrome, and Pearson's syndrome [4].

Nuclear DNA-related mutations have been found in genes directly or indirectly related to the RC, including, among others, (i) proteins involved in mtDNA maintenance and/or replication machinery; (ii) structural subunits of the RC complexes; (iii) assembly factors of the respiratory complexes; and (iv) components of the translation apparatus [5].

## Pathways to therapy

Currently, there is no treatment for mitochondrial diseases [6]. In the last few years, however, several potential therapeutic approaches have been proposed, and for some of them proof of efficacy has been provided in animal models, and testing their efficacy in humans is much awaited. These can be divided into two categories (Table 1): (i) those acting on common pathways and thus, in principle, applicable to several disorders and (ii) those tailored to a specific disease. The first category includes the stimulation of mitochondrial biogenesis, the improvement of respiration efficiency by shaping the cristae, the bypass OxPhos defects by using xenogenes [e.g. alternative oxidases (AOX) bypassing defects in cIII and cIV], and the use of antioxidants and other compounds to scavenge toxic metabolites. The second group includes Adeno-associated viral (AAV)-mediated gene therapy approaches aimed at re-expressing the wild-type gene or other therapeutic genes (e.g. endonucleases to shift the heteroplasmy) in targeted tissues. Here, I will briefly review these strategies (Table 2; for a more detailed review see ref. [7]). I will also summarize recent clinical trials, and the difficulties and challenges of translating a proof-of-principle experiment into clinics. Mitochondrial replacement therapy is discussed elsewhere [8].

**Table 1:** Overview of the therapeutic strategies for primary mitochondrial diseases

	Advantages	Disadvantages	Examples
General strategies	Wide applicability Potentially cost-effective Address common pathomechanisms	Off-target effects	Activation of mitochondrial biogenesis Regulating execution pathways (apoptosis, autophagy, and fission/fusion) Shaping mitochondrial cristae Bypass of RC defects by using xenogenes Use of dNTPs
Tailored strategies	Targeted for a single disease Potentially highly effective	Limited to a single/few conditions Expensive	AAV-mediated gene replacement Selective elimination of mutant mtDNA by ZNF or TALE nucleases

## Non-tailored strategies

### Nucleotide metabolism

Supplementation of deoxyribonucleosides has been used quite successfully in mouse models of mtDNA instability syndromes. This approach was highly effective in improving the biochemical and/or clinical defect *in vivo* in the *Tymp*^−/−^ mouse [9], a model of mitochondrial neuro-gastro-intestinal encephalomyopathy (MNGIE), and in the *Tk2*^−/−^ mouse, characterized by early-onset fatal encephalomyopathy [10]. *Tymp* encodes the cytosolic thymidine phosphorylase, which catalyzes the first step of thymidine and deoxyuridine catabolism; *Tk2* encodes mitochondrial thymidine kinase, which phosphorylates thymidine and deoxycytidine pyrimidine nucleosides to generate deoxythymidine monophosphate (dTMP) and deoxycytidine monophosphate (dCMP). Mutations in either enzymes lead to nucleotide imbalance and mtDNA instability, which can be rescued by the supplementation of deoxycytidine or tetrahydrouridine [9], an inhibitor of cytidine deaminase, in the case of the *Tymp*^−/−^ mouse model, and by dCMP + dTMP in the case of the *Tk2*^−/−^ mouse model [10].

### Stimulation of mitochondrial biogenesis

Bioenergetic defects and reduced ATP synthesis are key features of mitochondrial diseases and increasing mitochondrial mass or activity can thus be beneficial. The transcriptional co-activator peroxisome proliferator-activated receptor-γ1 (PGC1) α is the master regulator of mitochondrial biogenesis. PGC1α interacts with and increases the activity of several transcription factors, including the nuclear respiratory factors (NRF1 and 2), which, in turn, control the expression of OxPhos-related genes, and the peroxisomal proliferator activator receptors (PPARs) α, β, and γ, which control the expression of genes related to fatty acids oxidation [11]. In addition, PGC1α is activated by either deacetylation by Sirtuin 1 (Sirt1) or phosphorylation by AMP-dependent kinase (AMPK), both of which can be modulated pharmacologically [12]. For instance, AMPK is activated by AICAR, an adenosine monophosphate analog, whereas Sirt1 is activated by increasing cellular levels of NAD^+^, a co-substrate in the deacetylation reaction. This latter effect can be achieved by (i) providing NAD^+^ precursors and (ii) inhibiting NAD^+^ consuming enzymes such as the poly(ADP) ribosyl-polymerase 1. The administration of AICAR, nicotinamide riboside (NR), a NAD^+^ precursor, or PARP inhibitors was able to robustly induce mitochondrial biogenesis and ameliorate the clinical phenotype of two mouse models of mitochondrial myopathy [13–15]. NR seems to be particularly attractive for testing in patients, because it effectively enhances Sirt1 activity and mitochondrial biogenesis but lacks the unwanted effects of other components of vitamin B3. Nicotinic acid, for instance, although effectively increasing mitochondrial biogenesis, induces flushing by activation of GPR109A receptor, which is not stimulated by NR [16], while nicotinamide has been reported as an inhibitor of histone deacetylases, including sirtuins [17].

Finally, bezafibrate, a pan-PPAR agonist, has also been used to stimulate PGC1α leading to a remarkable clinical amelioration of a mouse model of severe cIV deficiency [18]. These results, however, were not confirmed in later studies on different mouse models [14,19], although the reasons for these discrepancies are unclear.

Overall, there is accumulating evidence in mouse models that boosting mitochondrial biogenesis can be an effective treatment for many mitochondrial diseases, independently of their genetic cause.

### Improving mitochondrial shape

Opa1 is a dynamin-like GTPase of the inner membrane playing a central role in cristae morphology. In humans, eight isoforms are generated by alternative splicing and processed by proteolytic cleavage by the two iAAA proteases, YME1 and OMA1, to form long (L-) and short (S-) forms, respectively. Increasing the expression of L-Opa1 improves respiration efficiency by increasing supercomplexes assembly [20] and protects *in vivo* from many insults such as ischemia–reperfusion, denervation-induced muscle atrophy, and OxPhos deficiency [21,22]. Accordingly, the up-regulation of L-Opa1 by deleting Oma1 delays neuronal loss and prolongs lifespan of prohibitin 2 knockout mouse [23]. Interestingly, a significant correction of mitochondrial ultrastructure in the same pathological conditions, and independently of the genetic cause, has been obtained by using Szeto-Schiller (SS) peptides [24]. These are tripeptides able to penetrate cells and to accumulate in mitochondria, where they bind cardiolipin, a lipidic component of the inner mitochondrial membrane with an important role in regulating the RC activity and in shaping mitochondrial cristae. Although the mechanism of SS peptides is poorly understood, cardiolipin modulates Opa1 activity and oligomerization [25], and they may actually modulate Opa1.

### Bypassing RC defects

Xenogenes, single-peptide enzymes derived from yeast or low eukaryotes, have been used to bypass the block of the RC due to defects in specific complexes in cellular and *Drosophila* models. The rationale for using these non-proton-pumping enzymes is that they should re-establish the electron flow, thus reducing the accumulation of reduced intermediates and ROS production, and increase ATP production by allowing proton pumping at the non-affected complexes. The NADH reductase (Ndi1), which in the yeast *Saccharomyces cerevisiae* transfers electrons from NADH to coenzyme Q (CoQ), has been used to bypass cI defects [26,27]. Similarly, AOX, which in various organisms transfers electrons from CoQ to molecular oxygen, has been used to bypass cIII and IV defects [28,29]. A transgenic mouse overexpressing *AOX* has been produced and did not show any gross abnormality [30], but the possibility to use AOX to bypass OxPhos defects *in vivo* in mammals has not yet been demonstrated.

## Disease-tailored strategies

AAV vectors are currently the most widely used vectors for gene therapy in humans because of several advantages, including the fact that they remain episomic, thus reducing the risk of insertional mutagenesis [31] and that an ever-expanding number of natural and engineered serotypes targeting different tissues has been described [32]. Integration of natural adeno-associated viruses into oncogenes, such as cyclin A2, and telomerase reverse transcriptase, has recently been reported and associated with hepatocellular carcinomas, although no such association has been so far reported for recombinant AAV vectors [33]. AAV's main limitations are related to their limited cloning capacity (no more than 4.7 kb should be included between ITRs), and the difficulty to target more tissues at the same time, which is a critical point for multisystem diseases (such as, in many cases, mitochondrial diseases).

Therapies with AAVs can be aimed at expressing either the wild-type form of mutated genes or other therapeutic genes (e.g. xenogenes).

### Gene replacement therapies

Hepatotropic AAV2/8 serotype was successfully used to express the wild-type form of mitochondrial sulfide di-oxygenase *Ethe1* in the liver of *Ethe1*^−/−^ mice, a model of ethylmalonic encephalopathy (EE), a highly severe mitochondrial disease due to impaired disposal of toxic hydrogen sulfide (H_2_S) and a poison for cytochrome *c* oxidase [34]. AAV2/8-*Ethe1* fully rescued enzyme activity, leading to efficient clearance of H_2_S from the bloodstream with a significant recovery of the profound cIV deficiency in the tissues and a striking prolongation of the lifespan [34]. The present study demonstrated that the selective re-expression of the missing gene into the liver was sufficient to induce a significant amelioration of the clinical phenotype in the mouse model, thus paving the way for using liver transplant for EE. The first child affected by EE was transplanted in Rome, Italy [35]. Eight months after the liver transplant, spectacular neurological improvement and achievements in psychomotor development were observed, accompanied by a remarkable amelioration of biochemical abnormalities.

Similarly, AAV2/8 has been used to treat the *Tymp*^−/−^ mouse model of the MNGIE disease [36], suggesting that gene therapy or liver transplant can be valuable options also for this disorder. An intravenous injection of AAV2/8 particles expressing human wild-type *TYMP* normalized dCTP and dTTP levels in plasma and tissues for up to 8 months of age. Finally, AAV2/8 was also successfully used to correct the liver-specific mtDNA depletion and to prevent ketogenic diet-induced cirrhosis in *MPV17*^−/−^ mice [37].

Although the AAV-based therapies summarized above were very effective in mice, extremely high costs for the production of the viral stocks and the rarity of the diseases prevented so far their application to the humans.

### Shifting heteroplasmy

Mitochondrially targeted restriction endonucleases have been used to shift heteroplasmy levels in cell lines with mutations in mtDNA and heteroplasmic mice [38,39]. This approach can, however, be used only when a suitable restriction site is introduced by the mutation, as in the case of the NARP mutation, which creates a SmaI restriction site. However, the introduction of TALE and zinc finger nucleases (TALEN and ZFN) allowed to bypass this limitation by addressing an unspecific restriction enzyme (FokI) to specific sites in the genome through the assembly of appropriate ZFN or TALE modules [40,41]. The main limitation of this approaches is that they both require quite large constructs not easily fitted into AAV vectors.

## From bench to bedside

### Overview on the clinical trials

Vitamins and food supplements (including CoQ, vitamins A and E, and lipoic acid) are normally used as a supportive therapy for mitochondrial disease [6], but no one can modify the disease course. Intrinsic difficulties, including the small cohorts of homogeneous patients available, the limited amount of data on the natural history of the diseases, and poor predictability of the outcome due to high variability, prevent solid trial design. However, several clinical trials, either open-label or randomized double-blind, are currently underway or have been recently completed (Table 3) [42], but the outcome is often unclear because the results have never been reported. The majority of the trials are focused on the use of antioxidants, especially on patients affected by LHON and MELAS, which offer rather big cohorts. For instance, EPI-743, a *para*-benzoquinone analog, is being tested on different types of mitochondrial diseases, including LHON and LS, while a trial on Pearson's disease has been terminated for unclear reasons. KH176, a derivative of the antioxidant Trolox, is being tested on MELAS patients. RTA408, a triterpenoid compound increasing antioxidant defenses by activating the nuclear factor erythroid 2-related factor 2 (Nrf2), is being tested on myopathic patients. Notably, in all these cases no preclinical data on animal models of mitochondrial disease have been collected to support the trial. CoQ_10_ and idebenone, a quinone analog of CoQ_10_, are among the very few cases, in which rather extensive studies have been carried out in patients so far. Idebenone was shown to ameliorate the rate of recovery in LHON patients with discordant visual acuity, especially when treated early in the disease course [43], and has been recently approved by EU for the treatment of this disease. Contrariwise, CoQ_10_, which is effective in some patients with the rare congenital CoQ_10_ deficiency, was shown to have little effect on other mitochondrial diseases [44].[Table bst-2016-0085TB3]

**Table 2:** Experimental therapies for mitochondrial diseases

Targeted pathway	Compounds	References
Nucleotide metabolism	• dCMP or tetrahydrouridine (inhibitor of cytidine deaminase)	[9]
	• dCMP + dTMP	[10]
PGC1α-dependent mitochondrial biogenesis	• AICAR (via AMPK)	[14]
	• Bezafibrate (via PPARs)	[18]
	• NR (via Sirt1) or PARP inhibitors	[13]
Mitochondrial shaping	• Increasing L-Opa1	[21]
	• Inhibition of Oma1	[23]
	• SS peptides	[24]
Bypassing OxPhos defects	• Ndi1 (bypass for cI defects)	[26,27]
	• AOX (bypass for cIII/cIV defects)	[28,29]
Shifting heteroplasmy	• Restriction endonucleases	[38,39]
	• ZNF nucleases	[40]
	• TALE nucleases	[41]
Elimination of toxic compounds	• AAV-mediated gene therapy	[34,36]
	• Liver transplant	[35]

**Table 3 bst-2016-0085TB3:** Examples of the clinical trials currently open or completed for mitochondrial diseases

Treatment	Disease	Trial number	Design	Target of intervention	Outcome
Currently open					
EPI-743	Metabolism or mitochondrial disorders	NCT01642056	Randomized, double-blind	ROS	Ongoing
Bezafibrate	Mitochondrial myopathy	NCT02398201	Open-label	Mitochondrial biogenesis	Ongoing
RTA 408	Mitochondrial myopathy	NCT02255422	Randomized, double- blind	ROS/NRF2	Ongoing
KH176	MELAS	NCT02544217	Randomized, double-blind	ROS	Ongoing
scAAV2-ND4	LHON	NCT02161380	Open-label	ND4	Ongoing
Completed					
Ketones	MELAS	NCT01252979	Open-label	Heteroplasmy	N/A
l-Arginine	MELAS	NCT01603446	Open-label	Nitric oxide	Improvement in aerobic capacity and muscle metabolism
Idebenone	LHON	NCT00747487	Randomized, double-blind	ROS	No recovery in visual acuity, but improvements in secondary end points (e.g. changes in visual acuity of the best eye at baseline)
Coenzyme Q10	Mitochondrial disease	NCT00432744	Randomized, double-blind	ROS	N/A
MTP-131	Mitochondrial myopathy	NCT02367014	Randomized, double-blind	Cardiolipin	N/A

Other compounds, with different mechanisms of action under clinical trials, include bezafibrate, MTP-131, ketones, and l-arginine [42]. In spite of the contradictory results in mice, a clinical trial with bezafibrate is recruiting patients. This was prompted by an increase in mitochondrial content observed in the skeletal muscle of bezafibrate-treated patients with carnitine palmitoyl transferase II defects [45]. A clinical trial with MTP-131 on patients with mitochondrial myopathies has been completed, but the results are not available yet. Ketones were shown to shift heteroplasmy in cellular models carrying mutations in mtDNA [46] and were tested on MELAS patients, but also in this case no results were reported. l-Arginine, a donor of nitric oxide thus acting on vessels tone, induced an improvement in aerobic capacity and muscle metabolism in MELAS patients [47].

Two clinical trials are being carried out using AAV vectors to allotopically express mitochondrial *ND4* in LHON patients. However, it is still highly debated if allotopically expressed proteins are really imported into the mitochondria and integrated into functionally active complexes [48].

### Conclusions

Mitochondrial medicine is experiencing a period of vibrant development. Several strategies have been proposed and some proved to be efficient in cell or animal models, but their application into the clinics is still challenging. Although the need for high-quality clinical trials has been repeatedly invoked [49], the transfer of preclinical studies into clinics is far from being a linear and easy process. More extensive collaborations between basic research laboratories, pharmacology experts, and industrial partners will be needed in order to tackle these problems and move the field into a new era.

## Abbreviations

AAV, adeno-associated viral; AICAR, 5-aminoimidazole-4-carboxamide ribonucleotide; AMPK, AMP-dependent kinase; AOX, alternative oxidases; CoQ, coenzyme Q; dCMP, deoxycytidine monophosphate; dTMP, deoxythymidine monophosphate; EE, ethylmalonic encephalopathy; GPR109A, G-protein coupled receptor 109A; H_2_S, hydrogen sulfide; ITR, inverted terminal repeat; LHON, Leber's hereditary optic neuropathy; LS, Leigh syndrome; MELAS, mitochondrial encephalomyopathy with lactic acidosis and stroke-like episodes; MERRF, myoclonic epilepsy with ragged red fibers; MNGIE, mitochondrial neuro-gastro-intestinal encephalomyopathy; mtDNA, mitochondrial DNA; MTP-131, mitochondria-targeted peptide 131; NARP, neurogenic weakness, ataxia, and retinitis pigmentosa; NR, nicotinamide riboside; Nrf2, nuclear factor erythroid 2-related factor 2; Opa1, optic atrophy 1; OxPhos, oxidative phosphorylation; PGC1, proliferator-activated receptor-γ1; PPAR, peroxisomal proliferator activator receptors; RC, respiratory chain; ROS, reactive oxygen species; Sirt1, Sirtuin 1; SS, Szeto-Schiller.

## Funding

This work was supported by the core grant from the MRC to Mitochondrial Biology Unit and by the grant [GR-2010-2306-756] from the Italian Ministry of Health (to C.V.).

## References

[1] Wallace D.C. (2005) A mitochondrial paradigm of metabolic and degenerative diseases, aging, and cancer: a dawn for evolutionary medicine. Annu. Rev. Genet. 39, 359–407 doi:10.1146/annurev.genet.39.110304.095751 16285865 PMC2821041

[2] Walker J.E. (2013) The ATP synthase: the understood, the uncertain and the unknown. Biochem. Soc. Trans. 41, 1–16 doi:10.1042/BST20110773 23356252

[3] Zeviani M. and Di Donato S. (2004) Mitochondrial disorders. Brain 127, 2153–2172 doi:10.1093/brain/awh259 15358637

[4] Chinnery P.F. (2015) Mitochondrial disease in adults: what's old and what's new? EMBO Mol. Med. 7, 1503–1512 doi:10.15252/emmm.201505079 26612854 PMC4693502

[5] Koopman W.J.H., Willems P.H.G.M. and Smeitink J.A.M. (2012) Monogenic mitochondrial disorders. N. Engl. J. Med. 366, 1132–1141 doi:10.1056/NEJMra1012478 22435372

[6] Pfeffer G., Majamaa K., Turnbull D.M., Thorburn D. and Chinnery P.F. (2012) Treatment for mitochondrial disorders. Cochrane Database Syst. Rev. CD004426 doi:10.1002/14651858.CD004426.pub3 22513923 PMC7201312

[7] Viscomi C., Bottani E. and Zeviani M. (2015) Emerging concepts in the therapy of mitochondrial disease. Biochim. Biophys. ActaBioenerg. 1847, 544–557 doi:10.1016/j.bbabio.2015.03.001 25766847

[8] Richardson J., Irving L., Hyslop L.A., Choudhary M., Murdoch A., Turnbull D.M. et al. (2015) Concise reviews: assisted reproductive technologies to prevent transmission of mitochondrial DNA disease. Stem Cells 33, 639–645 doi:10.1002/stem.1887 25377180 PMC4359624

[9] Camara Y., Gonzalez-Vioque E., Scarpelli M., Torres-Torronteras J., Caballero A., Hirano M. et al. (2014) Administration of deoxyribonucleosides or inhibition of their catabolism as a pharmacological approach for mitochondrial DNA depletion syndrome. Hum. Mol. Genet. 23, 2459–2467 doi:10.1093/hmg/ddt641 24362886 PMC6281351

[10] Garone C., Garcia-Diaz B., Emmanuele V., Lopez L.C., Tadesse S., Akman H.O. et al. (2014) Deoxypyrimidine monophosphate bypass therapy for thymidine kinase 2 deficiency. EMBO Mol. Med. 6, 1016–1027 doi:10.15252/emmm.201404092 24968719 PMC4154130

[11] Scarpulla R.C. (2008) Transcriptional paradigms in mammalian mitochondrial biogenesis and function. Physiol. Rev. 88, 611–638 doi:10.1152/physrev.00025.2007 18391175

[12] Puigserver P. and Spiegelman B.M. (2003) Peroxisome proliferator-activated receptor-γ coactivator 1α (PGC-1α): transcriptional coactivator and metabolic regulator. Endocr. Rev. 24, 78–90 doi:10.1210/er.2002-0012 12588810

[13] Cerutti R., Pirinen E., Lamperti C., Marchet S., Sauve A.A., Li W. et al. (2014) NAD^+^-dependent activation of Satirt1 corrects the phenotype in a mouse model of mitochondrial disease. Cell Metab. 19, 1042–1049 doi:10.1016/j.cmet.2014.04.001 PMC4051987 24814483

[14] Viscomi C., Bottani E., Civiletto G., Cerutti R., Moggio M., Fagiolari G. et al. (2011) In vivo correction of COX deficiency by activation of the AMPK/PGC-1α axis. Cell Metab. 14, 80–90 doi:10.1016/j.cmet.2011.04.011 21723506 PMC3130927

[15] Khan N.A., Auranen M., Paetau I., Pirinen E., Euro L., Forsström S. et al. (2014) Effective treatment of mitochondrial myopathy by nicotinamide riboside, a vitamin B3. EMBO Mol. Med. 6, 721–731 doi:10.1002/emmm.201403943 24711540 PMC4203351

[16] van de Weijer T., Phielix E., Bilet L., Williams E.G., Ropelle E.R., Bierwagen A. et al. (2015) Evidence for a direct effect of the NAD^+^ precursor acipimox on muscle mitochondrial function in humans. Diabetes 64, 1193–1201 doi:10.2337/db14-0667 25352640 PMC4375076

[17] Libri V., Yandim C., Athanasopoulos S., Loyse N., Natisvili T., Law P.P. et al. (2014) Epigenetic and neurological effects and safety of high-dose nicotinamide in patients with Friedreich's ataxia: an exploratory, open-label, dose-escalation study. Lancet 384, 504–513 doi:10.1016/S0140-6736(14)60382-2 24794816

[18] Wenz T., Diaz F., Spiegelman B.M. and Moraes C.T. (2008) Activation of the PPAR/PGC-1α pathway prevents a bioenergetic deficit and effectively improves a mitochondrial myopathy phenotype. Cell Metab. 8, 249–256 doi:10.1016/j.cmet.2008.07.006 18762025 PMC2613643

[19] Yatsuga S. and Suomalainen A. (2012) Effect of bezafibrate treatment on late-onset mitochondrial myopathy in mice. Hum. Mol. Genet. 21, 526–535 doi:10.1093/hmg/ddr482 22012983

[20] Pernas L. and Scorrano L. (2016) Mito-morphosis: mitochondrial fusion, fission, and cristae remodeling as key mediators of cellular function. Annu. Rev. Physiol. 78, 505–531 doi:10.1146/annurev-physiol-021115-105011 26667075

[21] Civiletto G., Varanita T., Cerutti R., Gorletta T., Barbaro S., Marchet S. et al. (2015) Opa1 overexpression ameliorates the phenotype of two mitochondrial disease mouse models. Cell Metab. 21, 845–854 doi:10.1016/j.cmet.2015.04.016 26039449 PMC4457891

[22] Varanita T., Soriano M.E., Romanello V., Zaglia T., Quintana-Cabrera R., Semenzato M. et al. (2015) The OPA1-dependent mitochondrial cristae remodeling pathway controls atrophic, apoptotic, and ischemic tissue damage. Cell Metab. 21, 834–844 doi:10.1016/j.cmet.2015.05.007 26039448 PMC4457892

[23] Korwitz A., Merkwirth C., Richter-Dennerlein R., Tröder S.E., Sprenger H.-G., Quirós P.M. et al. (2016) Loss of OMA1 delays neurodegeneration by preventing stress-induced OPA1 processing in mitochondria. J. Cell Biol. 212, 157–166 doi:10.1083/jcb.201507022 26783299 PMC4738383

[24] Szeto H.H. and Birk A.V. (2014) Serendipity and the discovery of novel compounds that restore mitochondrial plasticity. Clin. Pharmacol. Ther. 96, 672–683 doi:10.1038/clpt.2014.174 25188726 PMC4267688

[25] DeVay R.M., Dominguez-Ramirez L., Lackner L.L., Hoppins S., Stahlberg H. and Nunnari J. (2009) Coassembly of Mgm1 isoforms requires cardiolipin and mediates mitochondrial inner membrane fusion. J. Cell Biol. 186, 793–803 doi:10.1083/jcb.200906098 19752025 PMC2753158

[26] Perales-Clemente E., Bayona-Bafaluy M.P., Perez-Martos A., Barrientos A., Fernandez-Silva P. and Enriquez J.A. (2008) Restoration of electron transport without proton pumping in mammalian mitochondria. Proc. Natl Acad. Sci. USA 105, 18735–18739 doi:10.1073/pnas.0810518105 19020091 PMC2585044

[27] Sanz A., Soikkeli M., Portero-Otin M., Wilson A., Kemppainen E., McIlroy G. et al. (2010) Expression of the yeast NADH dehydrogenase Ndi1 in *Drosophila* confers increased lifespan independently of dietary restriction. Proc. Natl Acad. Sci. USA 107, 9105–9110 doi:10.1073/pnas.0911539107 20435911 PMC2889079

[28] Dassa E.P., Dufour E., Gonçalves S., Paupe V., Hakkaart G.A.J., Jacobs H.T. et al. (2009) Expression of the alternative oxidase complements cytochrome *c* oxidase deficiency in human cells. EMBO Mol. Med. 1, 30–36 doi:10.1002/emmm.200900001 20049701 PMC3378104

[29] Fernandez-Ayala D.J.M., Sanz A., Vartiainen S., Kemppainen K.K., Babusiak M., Mustalahti E. et al. (2009) Expression of the *Ciona intestinalis* alternative oxidase (AOX) in *Drosophila* complements defects in mitochondrial oxidative phosphorylation. Cell Metab. 9, 449–460 doi:10.1016/j.cmet.2009.03.004 19416715

[30] El-Khoury R., Dufour E., Rak M., Ramanantsoa N., Grandchamp N., Csaba Z. et al. (2013) Alternative oxidase expression in the mouse enables bypassing cytochrome *c* oxidase blockade and limits mitochondrial ROS overproduction. PLoS Genet. 9, e1003182 doi:10.1371/journal.pgen.1003182 23300486 PMC3536694

[31] Mingozzi F. and High K.A. (2011) Therapeutic in vivo gene transfer for genetic disease using AAV: progress and challenges. Nat. Rev. Genet. 12, 341–355 doi:10.1038/nrg2988 21499295

[32] Lisowski L., Tay S.S. and Alexander I.E. (2015) Adeno-associated virus serotypes for gene therapeutics. Curr. Opin. Pharmacol. 24, 59–67 doi:10.1016/j.coph.2015.07.006 26291407

[33] Nault J.-C., Datta S., Imbeaud S., Franconi A., Mallet M., Couchy G. et al. (2015) Recurrent AAV2-related insertional mutagenesis in human hepatocellular carcinomas. Nat. Genet. 47, 1187–1193 doi:10.1038/ng.3389 26301494

[34] Di Meo I., Auricchio A., Lamperti C., Burlina A., Viscomi C. and Zeviani M. (2012) Effective AAV-mediated gene therapy in a mouse model of ethylmalonic encephalopathy. EMBO Mol. Med. 4, 1008–1014 doi:10.1002/emmm.201201433 22903887 PMC3491831

[35] Dionisi-Vici C., Diodato D., Torre G., Picca S., Pariante R., Giuseppe Picardo S. et al. (2016) Liver transplant in ethylmalonic encephalopathy: a new treatment for an otherwise fatal disease. Brain 139(Pt 4), 1045–1051 doi:10.1093/brain/aww013 26917598

[36] Torres-Torronteras J., Viscomi C., Cabrera-Pérez R., Cámara Y., Di Meo I., Barquinero J. et al. (2014) Gene therapy using a liver-targeted AAV vector restores nucleoside and nucleotide homeostasis in a murine model of MNGIE. Mol. Ther. doi:10.1038/mt.2014.6 PMC4015233 24448160

[37] Bottani E., Giordano C., Civiletto G., Di Meo I., Auricchio A., Ciusani E. et al. (2013) AAV-mediated liver-specific MPV17 expression restores mtDNA levels and prevents diet-induced liver failure. Mol. Ther. 22, 10–17 doi:10.1038/mt.2013.230 24247928 PMC3880585

[38] Bacman S.R., Williams S.L., Duan D. and Moraes C.T. (2012) Manipulation of mtDNA heteroplasmy in all striated muscles of newborn mice by AAV9-mediated delivery of a mitochondria-targeted restriction endonuclease. Gene Ther. 19, 1101–1106 doi:10.1038/gt.2011.196 22130448 PMC3410960

[39] Srivastava S. and Moraes C.T. (2001) Manipulating mitochondrial DNA heteroplasmy by a mitochondrially targeted restriction endonuclease. Hum. Mol. Genet. 10, 3093–3099 doi:10.1093/hmg/10.26.3093 11751691

[40] Bacman S.R., Williams S.L., Pinto M., Peralta S. and Moraes C.T. (2013) Specific elimination of mutant mitochondrial genomes in patient-derived cells by mitoTALENs. Nat. Med. 19, 1111–1113 doi:10.1038/nm.3261 23913125 PMC4153471

[41] Gammage P.A., Rorbach J., Vincent A.I., Rebar E.J. and Minczuk M. (2014) Mitochondrially targeted ZFNs for selective degradation of pathogenic mitochondrial genomes bearing large-scale deletions or point mutations. EMBO Mol. Med. 6, 458–466 doi:10.1002/emmm.201303672 24567072 PMC3992073

[42] Koopman W.J., Beyrath J., Fung C.-W., Koene S., Rodenburg R.J., Willems P.H. et al. (2016) Mitochondrial disorders in children: toward development of small-molecule treatment strategies. EMBO Mol. Med. 8, 311–327 doi:10.15252/emmm.201506131 26951622 PMC4818752

[43] Klopstock T., Yu-Wai-Man P., Dimitriadis K., Rouleau J., Heck S., Bailie M. et al. (2011) A randomized placebo-controlled trial of idebenone in Leber's hereditary optic neuropathy. Brain 134, 2677–2686 doi:10.1093/brain/awr170 21788663 PMC3170530

[44] Glover E.I., Martin J., Maher A., Thornhill R.E., Moran G.R. and Tarnopolsky M.A. (2010) A randomized trial of coenzyme Q10 in mitochondrial disorders. Muscle Nerve 42, 739–748 doi:10.1002/mus.21758 20886510

[45] Djouadi F. and Bastin J. (2011) Species differences in the effects of bezafibrate as a potential treatment of mitochondrial disorders. Cell Metab. 14, 715–716; author reply 717 doi:10.1016/j.cmet.2011.11.003 22152297

[46] Santra S., Gilkerson R.W., Davidson M. and Schon E.A. (2004) Ketogenic treatment reduces deleted mitochondrial DNAs in cultured human cells. Ann. Neurol. 56, 662–669 doi:10.1002/ana.20240 15389892

[47] Rodan L.H., Wells G.D., Banks L., Thompson S., Schneiderman J.E. and Tein I. (2015) l-Arginine affects aerobic capacity and muscle metabolism in MELAS (mitochondrial encephalomyopathy, lactic acidosis and stroke-like episodes) syndrome. PLoS ONE 10, e0127066 doi:10.1371/journal.pone.0127066 25993630 PMC4439047

[48] Perales-Clemente E., Fernandez-Silva P., Acin-Perez R., Perez-Martos A. and Enriquez J.A. (2011) Allotopic expression of mitochondrial-encoded genes in mammals: achieved goal, undemonstrated mechanism or impossible task? Nucleic Acids Res. 39, 225–234 doi:10.1093/nar/gkq769 20823090 PMC3017613

[49] Pfeffer G., Horvath R., Klopstock T., Mootha V.K., Suomalainen A., Koene S. et al. (2013) New treatments for mitochondrial disease—no time to drop our standards. Nat. Rev. Neurol. 9, 474–481 doi:10.1038/nrneurol.2013.129 23817350 PMC4967498

